# Effect of Foot Rehabilitation Exercises for Painful Flat Foot in a 20-Year-Old Female: A Case Study Analysis

**DOI:** 10.7759/cureus.59377

**Published:** 2024-04-30

**Authors:** Pradhyum D Kolhe, H V Sharath, Siddhi G Rathi, Deepali S Patil

**Affiliations:** 1 Department of Paediatric Physiotherapy, Ravi Nair Physiotherapy College, Datta Meghe Institute of Higher Education and Research (DU) Sawangi Meghe, Wardha, IND; 2 Department of Musculoskeletal Physiotherapy, Ravi Nair Physiotherapy College, Datta Meghe Institute of Higher Education and Research (DU) Sawangi Meghe, Wardha, IND

**Keywords:** rehabilitation, physical therapy, foot deformity, flatfoot, pes planus

## Abstract

Pes planus, commonly referred to as flatfoot, is a congenital foot deformity characterized by the descent of the medial longitudinal arch, resulting in reduced spring action and increased stress on the foot during ambulation. This condition, opposite to pes cavus, typically lacks symptomatic presentation despite its structural abnormality. This case report discusses a 20-year-old female presenting to the musculoskeletal department of physiotherapy with impaired gait attributed to developmental flatfeet and an underdeveloped heel on one foot since birth. Apart from these foot deformities, no other significant abnormalities were noted upon examination. Orthotic management and ongoing monitoring have been initiated to facilitate functional independence. The prognosis for the patient's gait impairment remains optimistic with continued rehabilitation efforts aimed at dispelling misconceptions and barriers surrounding the correction of flatfoot deformities. This report underscores the importance of comprehensive rehabilitation strategies in managing flatfoot conditions to optimize patient outcomes and quality of life.

## Introduction

Pes planus is also known as flatfoot. It comes from Latin word which means pes as foot and planus as flat or ground level. A foot abnormality that is the opposite of pes cavus [1]. Here, the medial longitudinal arch of the foot descends, resulting in a lack of spring action and increased stress on the entire foot with each step. It serves as an adaptive support base for the entire body, functions to dissipate the forces of weight-bearing and acts to store energy during the gait cycle [2]. The dysfunction of the arch complex typically does not present with symptoms; however, it can impact the biomechanics of the lower limbs and lumbar spine, leading to a higher likelihood of experiencing pain and injury. Occurrence among children exceeds 70% during the initial four to six years of life, yet it has been documented to decline to approximately 9% post the age of six [3].

Flatfoot is defined by the International Classification of Diseases, Ninth Revision, Clinical Modification (ICD-9-CM) codes [4]. Flat feet are commonly seen in orthopedic clinics and are usually functional and painless [5]. The etiology of flatfoot remains elusive at present. Numerous studies propose a correlation between the debilitation of the intrinsic muscles of the foot and the diverse abnormalities that impact the foot arch, including pes cavus and pes planus [6].

## Case presentation

**Figure 1:**
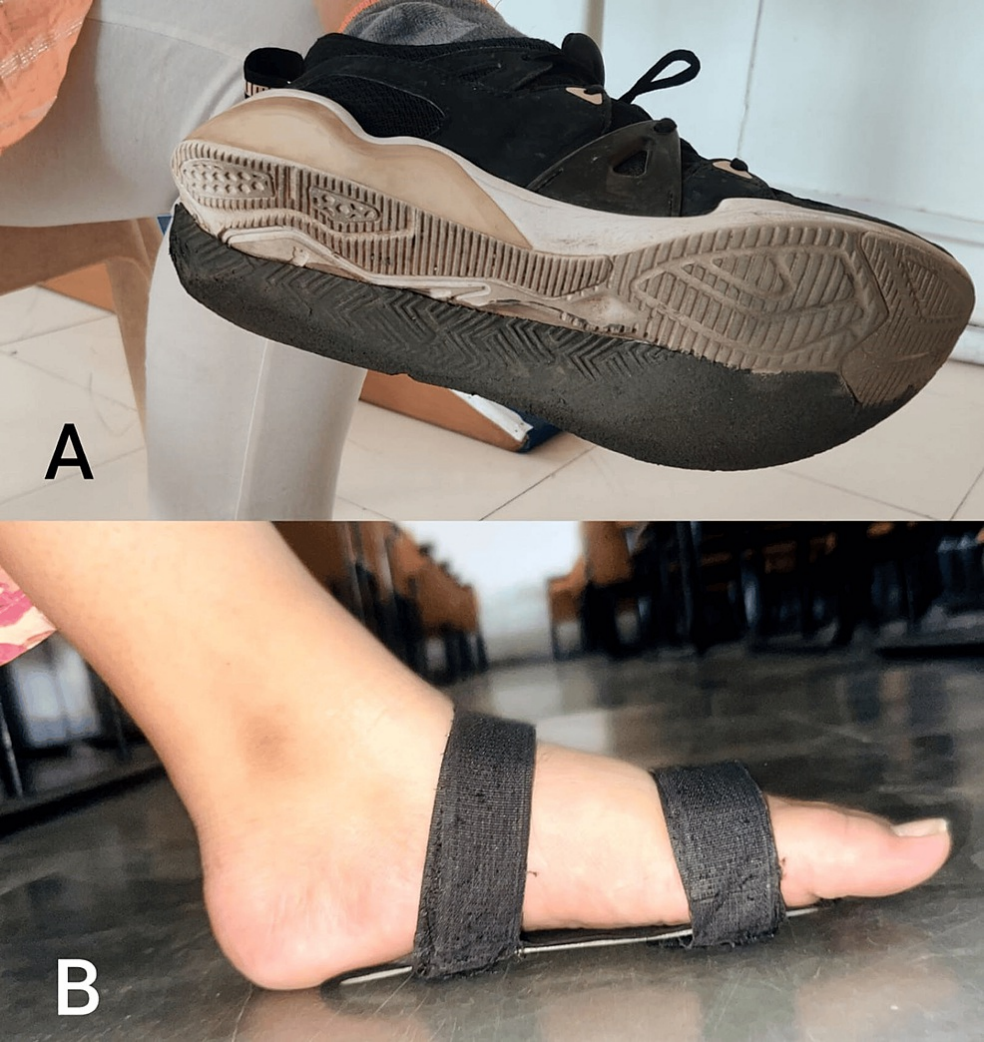
A- Implanted plates in shoes, B- Arch support splints

**Figure 2:**
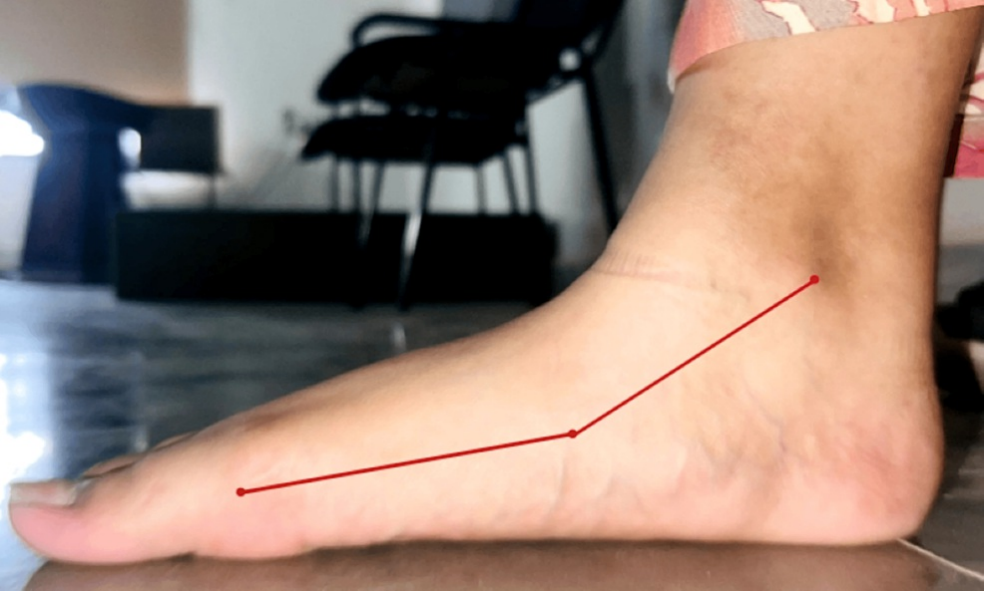
Fiess-line test

A 20 year female, arrived at the musculoskeletal department of physiotherapy with concerns about her walking difficulties. These difficulties were attributed to her developmental flatfeet and an underdeveloped heel on one foot, which she has had since birth. Despite these structural issues, her general examination showed no abnormalities, and she was able to maintain normal activity levels. Upon clinical examination, it was confirmed that she had bilateral flatfeet with decreased arches and an underdeveloped heel on one foot. She was utilizing orthotic interventions such as arch support splints for both feet (Figure 1A) and implanted plates in her shoes (Figure 1B) to assist with balance while walking. Flatfoot was confirmed through the Fiess line test, as illustrated in Figure 2.

On examination, manual muscle testing (MMT) (Table 1) was conducted on the patient with flatfoot before rehabilitation was given. It was observed that there was reduced strength in dorsiflexion and inversion movements compared to plantarflexion and eversion, indicative of possible muscle imbalances associated with the condition. Patient also complained of pain during strenuous activity. X-sens gait analysis is employed to examine and analyze gait patterns. However, apart from these foot deformities, no other significant abnormalities are noted. Neurologically, she displays normal muscle tone and strength in both lower extremities. Gait analysis indicates compensatory movements to adjust to the altered foot structure. With ongoing orthotic management and monitoring, her prognosis for maintaining functional independence remains positive.

**Table 1:** Pre and post-rehabilitation manual muscle testing (MMT) MMT- 0: No evidence of muscle contraction; 1: Trace contraction, but no movement; 2: Movement with gravity eliminated (passive range of motion); 3: Movement against gravity, but without resistance; 4: Movement against gravity and some resistance; 5: Normal strength, movement against full resistance

S.No.	Targeting Muscles	Pre-rehabilitation	Post-rehabilitation
1	Intrinsic muscles	1	4+
2	Abductor hallucis muscle (Toe abduction)	1	4+
3	Dorsiflexors	1	4+

Physiotherapy management 

Physiotherapy plays a pivotal role in the comprehensive management of patients with flatfoot, a condition characterized by the collapse or flattening of the arches of the feet. Through a tailored regimen of exercises and interventions, physiotherapists aim to alleviate symptoms, enhance foot strength, and improve overall biomechanics. The treatment protocol is typically designed for two months of duration with five days per week. It involves a combination of targeted exercises such as towel gathering, heel cord stretching, toe spreading, and posterior tibialis exercises. These exercises not only help in strengthening the intrinsic foot muscles but also promote flexibility and alignment of the foot structures. Additionally, incorporating barefoot walking or engaging in activities without supportive footwear allows for natural movement patterns, facilitating proprioceptive feedback and muscle activation. Table 2 displays a well-designed exercise protocol for foot rehabilitation. Under the guidance of a physiotherapist, patients embark on a journey of rehabilitation, gradually restoring function and stability to their feet. Through consistent dedication to the prescribed program, individuals with flatfoot can experience significant improvements in pain management, mobility, and overall quality of life, thereby highlighting the invaluable role of physiotherapy in the holistic management of this condition.

**Table 2:** Optimize exercise protocol for flatfoot rehabilitation

S.No.	Exercise program	Description of Exercise	Intensity
1	Scrunching exercise	Scrunching exercise that involves compressing a towel that is lying on the floor between the toes.	15 minutes/session
2	Achilles tendon stretching	One leg should be forward and slightly bent at the knee while facing a wall. inform the participants to place their other leg behind them and to keep both their heels flat on the ground. Asked to gently press your hips against the wall	Hold for 30 seconds, followed by a 30-second relaxation period, and then repeat once 3 sets of 10 repetitions
3	Toe abduction exercise	Placing both feet flat on the floor. Tell to abduct toes as widely as possible.	Hold out for 5 seconds and then relax for 2 seconds. 3 sets of 10 repetitions
4	Strengthening of Posterior Tibialis	For these the participant has to cross seat and tie Band of resistance around foot. On other end of band underneath to the other foot. Ankle has to be relaxed and tell to move foot upwards towards roof. Return to start position slowly	3 sets of 10 repetitions 5 days once a week for 60 days
5	Golf ball rolling	Determine which foot is afflicted. While sitting comfortably on a chair with both feet flat on the floor. Place the golf ball beneath the arch of the foot, then roll it back and forth under the arch with light pressure	Continue this rolling motion for approximately 2 minutes.
6	Bare foot walking	Participants were asked to go barefoot or told to wear either sock, slippers without any supports and standing activities like watching TV, playing, key is that they have stand on their feet	Bare foot walking for at-least 45 minutes with barefoot activity per day, 5 times a week

**Figure 3:**
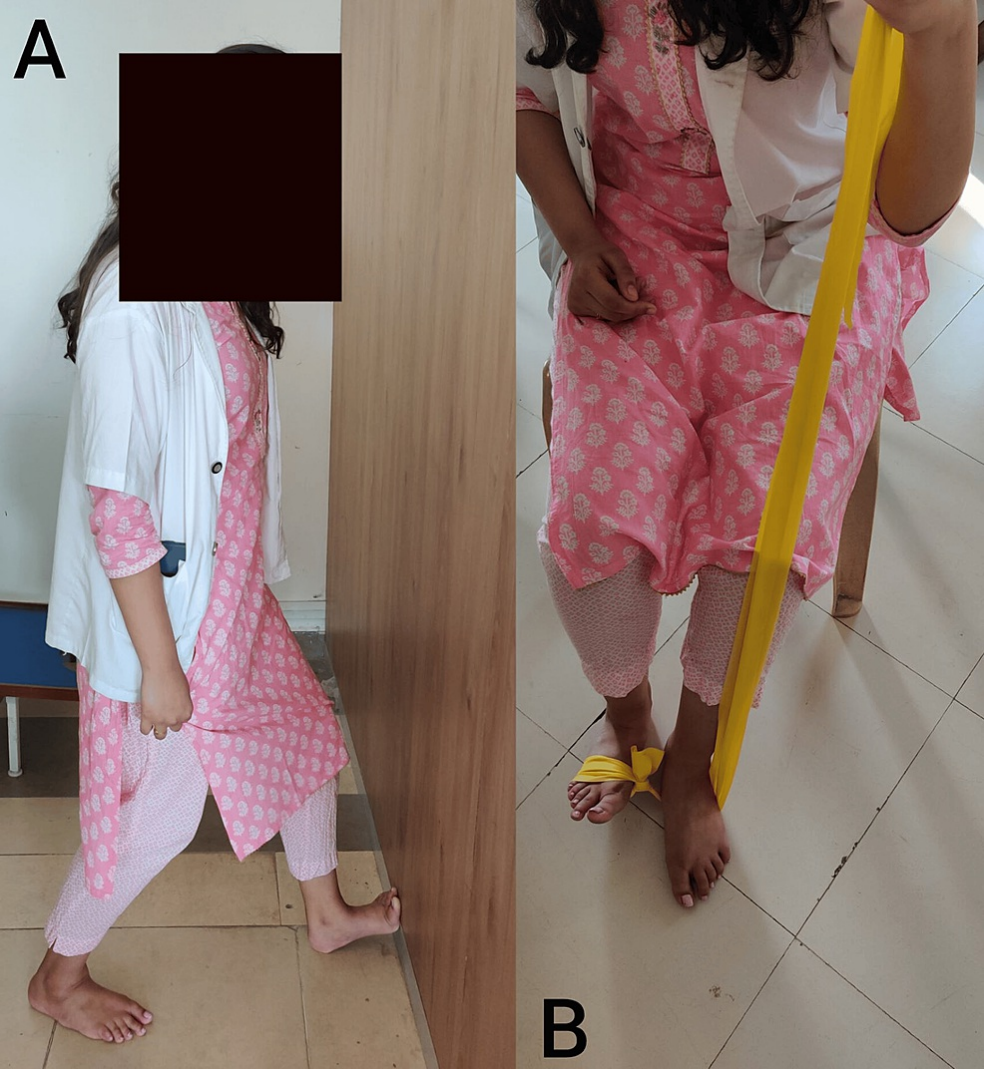
A- Achilles tendon stretching and B- Strengthening of Posterior Tibialis

Stand facing a wall with one leg forward and slightly bent at the knee. Place the other leg behind you, keeping both heels flat on the ground Gently press your hips against the wall (Figure 3A). Cross a resistance band around the foot of the leg that's behind you. Secure the other end of the band under the foot of the leg that's forward. Keep your ankle relaxed and slowly move your foot upwards towards the ceiling. Return to the start position slowly. Repeat this exercise, then switch legs. Note any differences in difficulty or discomfort between the two legs mentioned in Figure 3B. 

**Figure 4:**
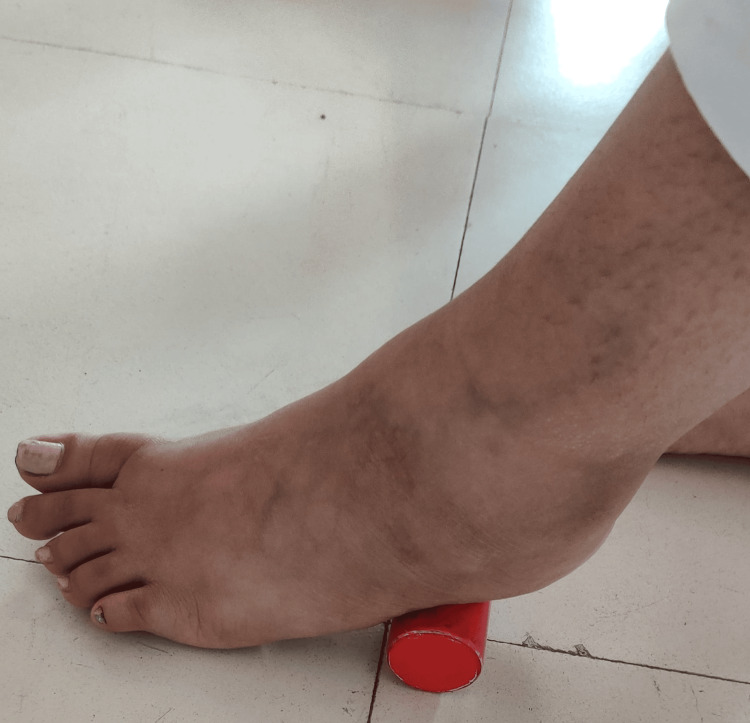
Golf ball rolling exercise

Sit comfortably on a chair with both feet flat on the floor. Place a golf ball beneath the arch of one foot. Apply light pressure and roll the golf ball back and forth under the arch of your foot. Continue rolling for a few minutes, adjusting pressure as needed. Switch to the other foot and repeat the process. Pay attention to any areas of tension or discomfort as mentioned in Figure 4.

Outcome measures, a visual analog scale is used to assess pain levels during strenuous activities of the patient, along with foot assessment scales taken such as Foot Function Index (FFI), Foot and Ankle Ability Measures (FAAM), Foot Posture Index (FPI), as mentioned in Table 3.

**Table 3:** Assessing scales for flatfoot FFI: 1: No pain; 2: Mild pain; 3: Moderate pain; 4: Severe pain; 5: Very severe pain; 6: Worst pain imaginable; 1: No difficulty; 2: Mild difficulty; 3: Moderate difficulty; 4: Severe difficulty; 5: Very severe difficulty; 6: So difficult unable; Activity limitation 1: None of the time; 2: A little of the time; 3: Some of the time; 4: Much of the time; 5: Most of the time; 6: All of the time VAS: 0: No pain; 10: Worst pain FAAM: Higher (100%) the score lesser the disability FFPI-6: Normal: 0 to +5; Pronated: +6 to +9, Highly Pronated +10: Supinated: -1 to -4, Highly supinated -5 to -12

S.No.	Assessing scales	Pre-rehabilitation (/n)	Post-rehabilitation (/n)
1	Foot Function Index (FFI)	Pain and stiffness: 5/6	Pain and stiffness: 2/6
		Difficulty: 4/6	Difficulty: 1/6
		Activity Limitation: 5/6	Activity Limitation: 1/6
2	Visual analogue scale (VAS)	07/10	02/10
3	Foot and Ankle Ability Measures (FAAM)	ADL subscale - 50%	ADL subscale- 97%
		Sports subscale- 50%	Sports subscale- 100%
4	Foot Posture Index (FPI-6)	06/12	-2/12

## Discussion

Pes planus is a prevalent condition marked by the collapse of the medial arch, abduction of the forefoot, internal rotation, plantar flexion of the talus, and eversion of the calcaneus [7]. Subotnick states that pes planus is observed in 20% of the overall population [8]. Khamis and Yizhar et al. noted that changes in foot structure can have an impact on adjacent body segments due to the interconnected nature of the body's structures, which can be likened to a chain mechanism [9]. The kinematic chain exercise demonstrated a significant impact on individuals suffering from flat feet, resulting in noticeable improvements in pain levels [10].

The present case has difficulty in walking due to flat feet and an underdeveloped heel highlights the importance of physiotherapy in treating such conditions. Physiotherapists play a vital role in dealing with the issues caused by flat feet by using specific exercises and treatments to reduce symptoms and enhance foot mechanics. The recommended two-month treatment plan, which includes exercises such as towel scrunches, heel stretches, toe spreads, and posterior tibialis exercises done five times a week, shows potential in strengthening the muscles in the feet and improving their flexibility and alignment. Encouraging activities without shoes also helps promote natural movements, which aids in providing feedback to the muscles and activating them.

The exercise plan provided offers a comprehensive approach to foot rehabilitation, highlighting the diverse aspects of physiotherapy in addressing concerns related to flat feet. Towel-gathering exercises, for instance, help strengthen the intrinsic foot muscles [11]. Meanwhile, heel cord stretching promotes flexibility in the Achilles tendon and calf muscles, essential for achieving proper foot alignment during gait [12]. Furthermore, incorporating exercises that target toe spreading and activate the posterior tibialis muscle aids in stabilizing and providing support to the arches of the foot. Strengthening the posterior tibialis muscle, in particular, is pivotal in preventing excessive pronation and supporting the medial longitudinal arch [13]. FFI is a self-administered survey utilized for evaluating how foot problems or injuries affect a person's capacity to carry out everyday tasks [14].

Visual analogue scale used to check pre- and post-rehabilitation pain level. FAAM is a questionnaire that individuals complete to assess their functional limitations and disabilities associated with foot and ankle conditions [15]. FPI is a clinical tool used to assess the static alignment of the foot and ankle complex. A cross-sectional investigation was carried out in order to assess and contrast the reliability and diagnostic precision of the FPI-6 and Clarke’s angle (CA) in identifying flexible flatfoot in adolescents aged 12 to 18 years, taking into account radiographic examination as the benchmark measure [16]. In their systematic review, Hara et al. analyzed the impacts of short-foot exercise (SFE) in contrast to foot orthosis or alternative forms of interventions [17]. Physiotherapy interventions focus on these particular muscle groups to address biomechanical abnormalities linked to flatfoot, ultimately relieving symptoms and enhancing functional outcomes [18-20].

## Conclusions

In this case study, the efficacy of foot rehabilitation exercises in managing painful flat foot in a 20-year-old female was investigated. The patient's presentation with foot pain and discomfort due to flat feet significantly impacted her daily functioning and quality of life. Through a structured rehabilitation program comprising targeted exercises, stretching, and orthotic intervention, notable improvements were observed in pain reduction, functional mobility, and foot posture. The implementation of a personalized exercise regimen focusing on intrinsic foot muscle strengthening, arch support enhancement, and proprioception improvement played a pivotal role in addressing the underlying biomechanical issues contributing to painful flat foot. Moreover, incorporating stretching exercises helped alleviate muscle tightness and enhance flexibility, augmenting the effectiveness of the rehabilitation program. This case study underscores the importance of tailored rehabilitation protocols tailored to the individual needs of patients with painful flat foot. By targeting biomechanical abnormalities, strengthening intrinsic foot muscles, and optimizing foot posture, clinicians can effectively alleviate symptoms, enhance functional outcomes, and improve overall quality of life for individuals with this condition. Further research and larger-scale studies are warranted to validate these findings and elucidate the long-term efficacy of foot rehabilitation interventions for painful flat foot. Ultimately, continuous studies on foot rehabilitation not only improve clinical procedures but also play a fundamental role in fostering overall wellness and reinstating functional autonomy for individuals.
